# Wide distribution of Mediterranean and African spotted fever agents and the first identification of Israeli spotted fever agent in ticks in Uganda

**DOI:** 10.1371/journal.pntd.0011273

**Published:** 2023-07-27

**Authors:** Wilfred Eneku, Bernard Erima, Anatoli Maranda Byaruhanga, Gladys Atim, Titus Tugume, Qouilazoni A. Ukuli, Hannah Kibuuka, Edison Mworozi, Christina Douglas, Jeffrey W. Koehler, Nora G. Cleary, Michael E. von Fricken, Robert Tweyongyere, Fred Wabwire-Mangen, Denis Karuhize Byarugaba

**Affiliations:** 1 Makerere University, College of Veterinary Medicine, Kampala, Uganda; 2 Makerere University Walter Reed Project, Kampala, Uganda; 3 Makerere University, College of Health Sciences, Kampala, Uganda; 4 Diagnostic Systems Division, USAMRIID, Fort Detrick, Maryland, United States of America; 5 Global and Community Health, George Mason University, Fairfax, Virginia, United States of America; 6 Makerere University, School of Public Health, Kampala, Uganda; Chengde Medical University, CHINA

## Abstract

*Rickettsia* microorganisms are causative agents of several neglected emerging infectious diseases in humans transmitted by arthropods including ticks. In this study, ticks were collected from four geographical regions of Uganda and pooled in sizes of 1–179 ticks based on location, tick species, life stage, host, and time of collection. Then, they were tested by real-time PCR for *Rickettsia* species with primers targeting *gltA*, *17kDa* and *omp*A genes, followed by Sanger sequencing of the *17kDa* and *ompA* genes. Of the 471 tick pools tested, 116 (24.6%) were positive for *Rickettsia* spp. by the *gltA* primers. The prevalence of *Rickettsia* varied by district with Gulu recording the highest (30.1%) followed by Luwero (28.1%) and Kasese had the lowest (14%). Tick pools from livestock (cattle, goats, sheep, and pigs) had the highest positivity rate, 26.9%, followed by vegetation, 23.1%, and pets (dogs and cats), 19.7%. Of 116 *gltA*-positive tick pools, 86 pools were positive using *17kDa* primers of which 48 purified PCR products were successfully sequenced. The predominant *Rickettsia* spp. identified was *R*. *africae* (n = 15) in four tick species, followed by *R*. *conorii* (n = 5) in three tick species (*Haemaphysalis elliptica*, *Rhipicephalus appendiculatus*, and *Rh*. *decoloratus*). *Rickettsia conorii* subsp. *israelensis* was detected in one tick pool. These findings indicate that multiple *Rickettsia* spp. capable of causing human illness are circulating in the four diverse geographical regions of Uganda including new strains previously known to occur in the Mediterranean region. Physicians should be informed about *Rickettsia* spp. as potential causes of acute febrile illnesses in these regions. Continued and expanded surveillance is essential to further identify and locate potential hotspots with *Rickettsia* spp. of concern.

## Author summary

Tick-borne rickettsioses are emerging infectious diseases of public health importance worldwide. Spotted fever rickettsioses (SFG) transmitted by ticks can cause mild to severe human illness depending on the pathogen species and co-morbidities. They present with non-specific symptoms making diagnosis challenging particularly in limited resource settings where there is a lack of effective diagnostic testing. Little is known about the prevalence of *Rickettsia* in Uganda. Using entomological and molecular tools, we surveyed and studied tick-borne SFG *Rickettsia* in five districts from four diverse eco-regions of Uganda. Overall, 24.6% (116/471) tick pools were positive for *Rickettsia* species. Sequencing the *17kDa* and *ompA* genes of *Rickettsia* revealed *R*. *africae* as the most common agent, followed by *R*. *conorii* and *R*. *conorii* subsp. *israelensis*. The findings indicate multiple *Rickettsia* spp. are circulating in the four geographically diverse regions of Uganda that can cause febrile illness in humans. Physicians should be aware of these agents as potential causes of febrile illness in these areas.

## Introduction

Arthropod vectors transmit many pathogens to humans including *Rickettsia* which is a bacteria responsible for multiple emerging infectious diseases globally [1–3]. Clinical presentations of rickettsioses differ by *Rickettsia* group; the Spotted Fever Group (SFG) transmitted by ticks and the Typhus Group (TG) transmitted by fleas and lice. Seroprevalence rates for *Rickettsia* spp. vary widely and range from 8–10% in the East African region and <1–37% worldwide [4–8]. These pathogens are increasingly associated with undifferentiated febrile illnesses in humans potentially resulting in severe illness and/or death [9–10]. Several cases of SFG rickettsioses have been reported in international travellers returning to their home countries, particularly from endemic regions in sub-Saharan Africa and southeast Asia [11–14]. Historically, these diseases have been poorly studied in sub-Saharan Africa where the largest burden of disease exists, particularly in indigenous populations [15].

Rickettsioses manifest with non-specific signs such as fever, severe headache, skin rash and general malaise, which is often misdiagnosed as other febrile illnesses or viral diseases. Confirmation of a rickettsial infection requires direct molecular detection or serological testing to detect antibodies, potentially leading to a false negative results if testing occurs too early in the bacterial infection before antibodies are generated [12,16–17]. Moreover, proper testing requires expensive equipment and reagents and relies on skilled laboratory technicians complicating the ability of countries with constrained resources to test for *Rickettsia* infections. Cases of febrile illnesses are often over diagnosed as malaria and later proven otherwise by more sensitive and specific PCR assays [18]. Distinguishing *Rickettsia* from other pathogenic agents early allows for timely treatment and informs any necessary public health measures.

Uganda is home to multiple medically relevant arthropod vectors including ticks, fleas, and mites. In two regions of the country, the northeast and southwest, diverse species of ticks have been recovered from both animals and the environment carrying medically relevant human and animal pathogens [19–20]. We recently reported the abundance and distribution of seven tick species of the *Rhipicephalus*, *Haemaphysalis*, *and Amblyomma* genera with rickettsial pathogens detected in the Ugandan cattle corridor [21]. Tick-borne *Rickettsia* spp., *R*. *africae*, *R*. *conorii*, and *R*. *massiliae*, of human relevance are prevalent in Uganda [22–24].

*Rickettsia africae* is the etiologic agent of African tick-bite fever (ABTF) transmitted predominantly by *Amblyomma* tick species. Infection with *R*. *africae* is the most common tick-borne bacterial zoonosis reported in travelers returning from sub-Saharan Africa [6,11]. *Rickettsia conorii*, mainly transmitted by *Rh*. *sanguineus* tick species, is endemic in the Mediterranean and causes Mediterranean spotted fever (MSF) [10]. Israeli spotted fever (ISF), a disease similar to but more severe than MSF, is caused by the subspecies *R*. *conorii israelensis*. *Rickettsia conorii israelensis* is also transmitted by *Rh*. *sanguineus* (sensu lato) [22]. Although these diseases are generally mild and manifest with the common characteristics of rickettsioses, infection often results in hospitalizations and delays the diagnosis of potentially co-infected febrile illnesses [6,22].

Tick population territories have changed significantly in the past decade largely due to anthropogenic and environmental changes resulting from climate change [10, 25–26]. An increase in densities of medically important vectors and pathogens is commonly associated with emergence of disease in humans, representing a major public health concern. This poses a risk to the 58% of Ugandans who derive their livelihoods through livestock keeping, predominantly kept on open grazing [27]. Limited knowledge about the ticks associated with SFG rickettsia transmission, their frequencies, and geographic range in Uganda creates challenges in designing appropriate control measures. While there is evidence of widespread *Rickettsia* spp. present throughout sub-Saharan Africa, there is limited data about the species and frequency in Ugandan ticks [21]. Therefore, it is essential to characterise ticks and their associated *Rickettsia* pathogens to better inform control strategies and contribute to our understanding of tick-borne diseases in Uganda.

## Materials and methods

### Study sites

**Fig 1:**
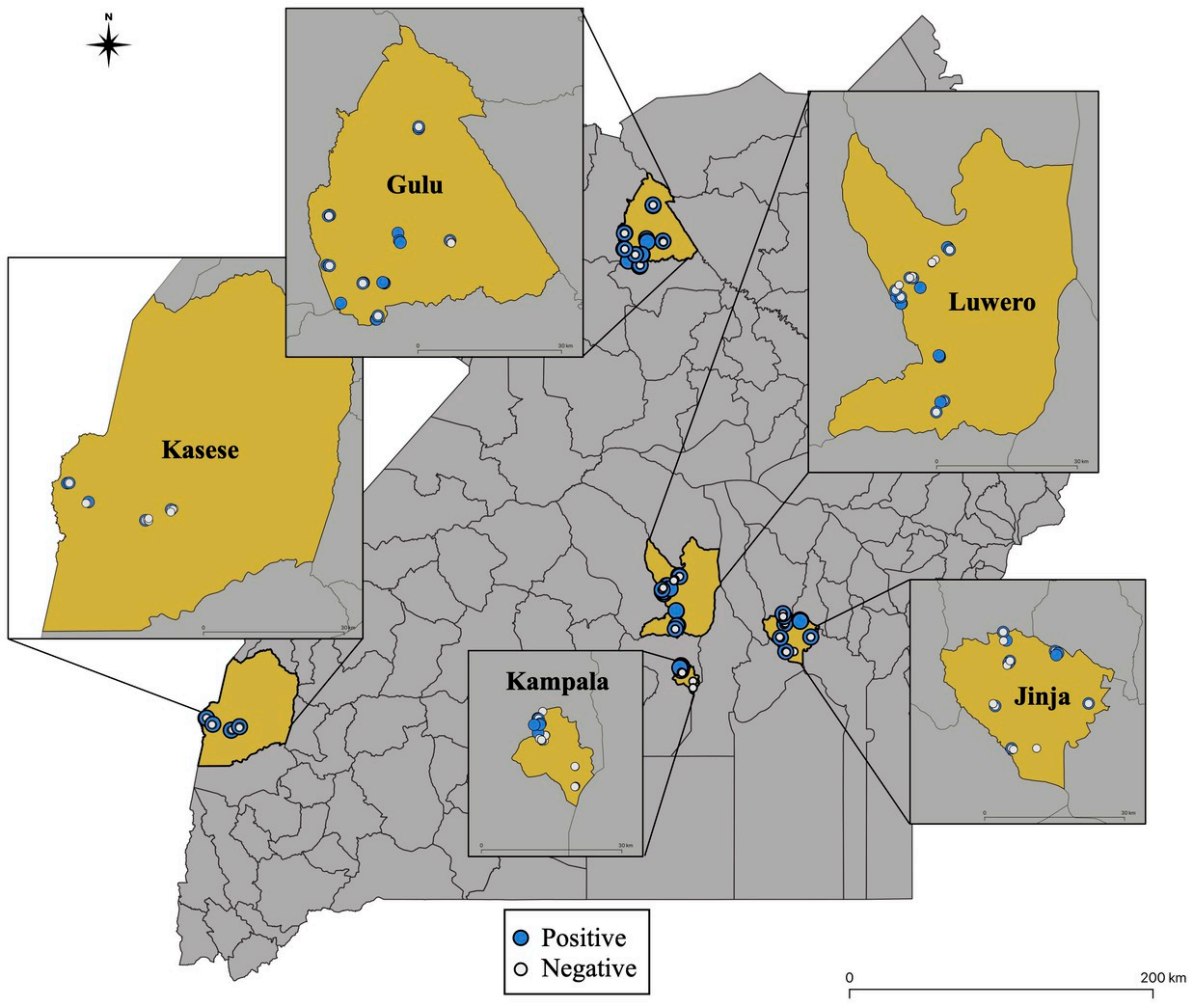
A map of Uganda indicating sites from where pools of ticks were collected and tested for *Rickettsia* spp. Those tested negative (white circle) and positive (blue circle) are indicated. This map was generated using QGIS 3.28 with the base map accessible at https://data.unhcr.org/en/documents/details/83043.

Ticks were collected from homesteads in five districts [Jinja (Eastern Uganda), Kampala (Capital of Uganda), Kasese (Western Uganda), Gulu (Northern Uganda) and Luwero (Central Uganda)] (Fig 1). The selected districts are considered major economic hubs in their respective regions and are geographically and culturally diverse, with high levels of economic heterogeneity. The source of the ticks were livestock (cattle, goats, sheep, pigs), companion animals (dogs and cats), chicken, and from the grass environment, collected between April 2017 and September 2018. Ticks collected from animals were picked with forceps and preserved in 70% ethanol. The ticks from vegetation were collected by dragging a 1m^2^ white flannel cloth on vegetation midmorning (after dew drop) around homesteads where ticks from animals were collected. Depending on the size of area available around the homesteads, an approximate area covering at least 100m^2^ (10 x10m or 100m long transect) were sampled as previously described [28–29]. Five transects about 2 meters apart were covered by a slow pace of dragging, checking the cloth every 15–20 paces. Ticks that attached to the cloth were picked with fine-tipped forceps (tweezer) and also preserved in 70% ethanol.

### Tick pools

Ticks were identified to the species level using morphologic taxonomic keys [30] under a stereomicroscope. A total of 5,790 ticks were sorted into 471 pools (1–179 ticks per pool, average 12.3) according to tick species, host, collection area, date of collection and developmental stage. There were 306 pools (5,408 ticks) from livestock, 138 pools (677 ticks) from companion animals, 1 pool (1 tick) from a chicken, and 26 pools (64 ticks) from vegetation. The pools were placed in Eppendorf tubes containing RNA later (Sigma Life Science, Darmstadt, Germany) and disrupted using sterile disposable pestles attached to a motorized grinder (HLD-12, Ryobi, China). Ticks were then homogenized by passing them through 20-gauge needles, with homogenate then stored at -80°C until DNA extraction.

### DNA extraction and PCR

Total DNA was extracted from the tick homogenates using the Qiagen DNeasy Blood and Tissue kit (Qiagen, Hilden, Germany), according to the manufacturer’s protocol. Every batch of samples were extracted alongside two positive and two negative controls. All 471 tick pool DNA samples were screened for SFG *Rickettsia* spp. with primers amplifying the 74-bp citrate synthase (*gltA*) gene as previously described [31–32]. The primers (CS-F (5-TCGCAAATGTTCACGGTACTTT-3) and CS-R (5-TCGTGCATTTCTTTCCATTGTG-3) were used with the Platinum Quantitative PCR SuperMix-UDG (ThermoFisher Scientific) PCR kit. Briefly, the qPCR conditions involved initial incubation of the reactions at 50°C for 2 min, then denaturation at 94°C for 2 min followed by 45 cycles of two-step amplification at 94°C for 15 sec and 60°C for 1 min in a 7500 Real-Time PCR System (Applied Biosystems, US). Only positive samples from the screening were subsequently tested for 115-bp segment of the 17kDa gene and ompA genes using the primers and methods previously described to confirm the initial PCR results [31]. For every run, two positive and two negative controls were set. The first two wells set were negative controls, followed by the templates (samples) and then the positive controls were set last (in the last wells). In between the templates, new pipette tips were used with regular changes of gloves. *Rickettsia conorii* DNA (provided by Walter Reed Army Institute of Research, Silver Spring, MD) was used as a positive control and ultrapure water as a negative control.

### Sequencing and phylogenetic analysis

A 539 base pair amplicon for *17kDa* and a 650 base pair amplicon for *ompA* gene was amplified as previously described [33] using Platinum taq (Thermo Fisher Scientific). Cycling conditions for *ompA* amplification were 95°C for 2 min and 45 cycles of 95°C for 30 sec, 42°C for 35 sec, and 60°C for 2 min. Cycling conditions for *17kDa* amplification were 95°C for 2 min and 45 cycles of 95°C for 30 sec, 57°C for 60 sec, and 72°C for 2 min. PCR products were resolved on a 2% agarose gel and purified using the QIAquick PCR Purification Kit (Qiagen). Samples were sequenced on the SeqStudio (Thermo Fisher Scientific) using the BigDye Terminator v3.1 Cycle Sequencing Kit (Thermo Fisher Scientific) according to the manufacturer’s recommendations. Forward and reverse reads were aligned using CLC Genomics Workbench (Qiagen) and a consensus sequence for each gene was generated for BLAST analysis. Sequences of *17kDa* and *ompA* genes and references from GenBank were imported and aligned in Geneious Prime 2022.11.0.14.1. The sequences were MAFFT aligned and exported to MEGA 10.2.6 [34] where maximum likelihood trees were created at 1,000 bootstrap iterations.

### Mapping

Descriptive maps showing the collection sites were created in QGIS 3.28 [35]. The Uganda district shapefile is available at https://data.unhcr.org/en/documents/details/83043.

### Statistical analysis

The probability of *Rickettsia* spp. detection from the pooled tick samples was estimated using detection rates; maximum likelihood estimation (MLE) and minimum infection rate (MIR) by collection district and tick species. Both MLE and MIR estimates and their corresponding confidence intervals were calculated accounting for individual pool sample sizes using the CDC’s Mosquito Surveillance Software (https://www.cdc.gov/westnile/resourcepages/mosqSurvSoft.html). A Pearson chi-squared test was used to detect any differences between the distributions of outcomes in different groups, with a p-value of <0.05 considered significant. Data were analyzed using STATA software, version 16.1 (StataCorp, College Station, TX).

## Results

### Distribution of tick species by collection sites

**Fig 2:**
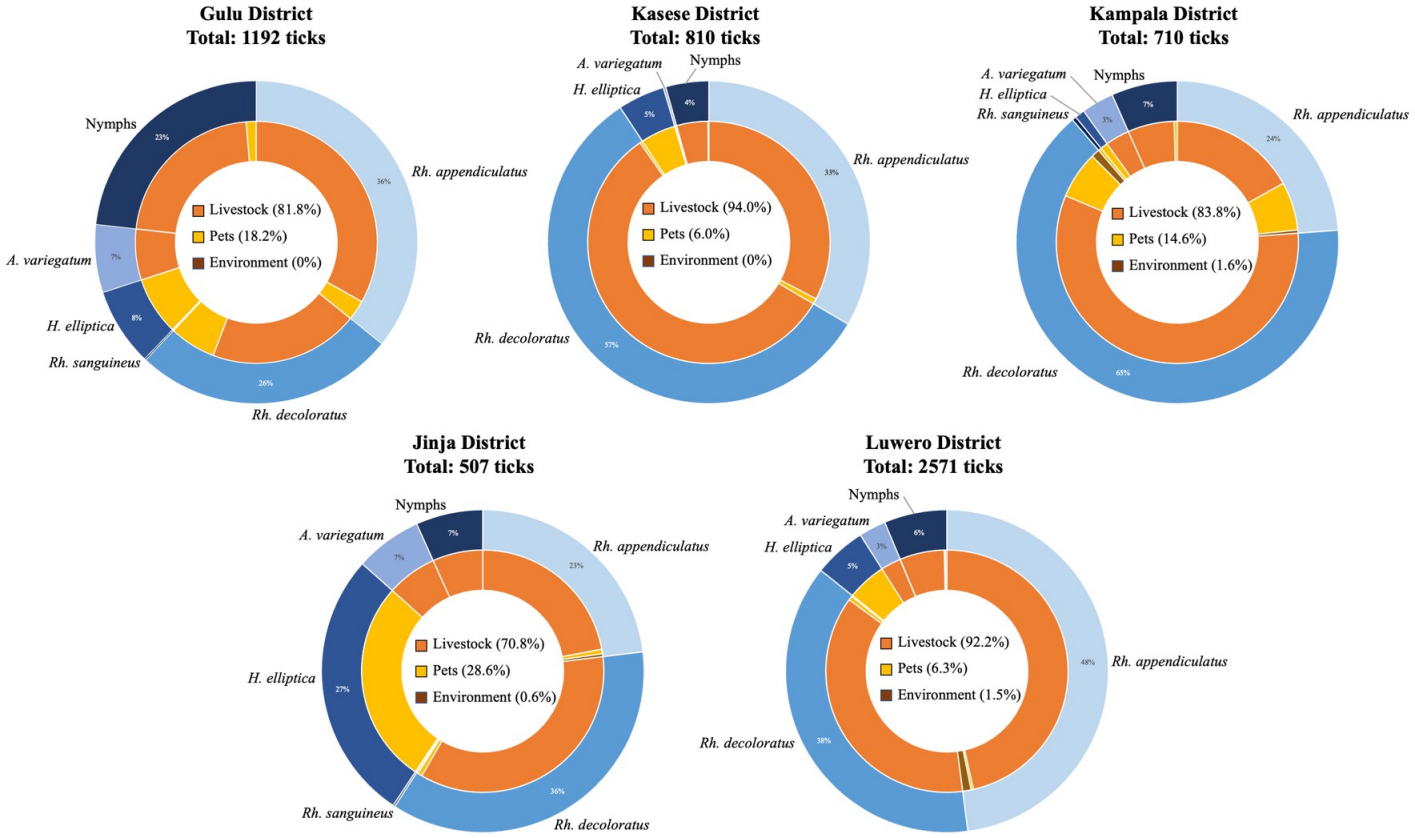
Distribution of ticks by collection district and host. The total number of ticks collected in each district is listed below the respective district name. The outer circle represents the percentage of each tick species from the respective district. The inner circle represents the host distribution of each respective tick species. The total host distribution by district is shown by the percentages in the middle. Livestock includes the chicken recorded from Gulu district. Labels for percentages less than or equal to 1 were excluded.

Five tick species were identified from the collections across the five districts. *Rhipicephalus* genera ticks accounted for over half of collections from each district. The most abundant tick species was *Rh*. *appendiculatus* which constituted 30.6% of all tick pools collected, followed by *Rh*. *decoloratus* (28.2%) and the least collected tick by pool count was *Rh*. *sanguineus* (0.8%). *Rhipicephalus sanguineus* was collected from dogs in three districts (Gulu, Jinja, and Kampala) whereas the other four species (*Rh*. *appendiculatus*, *Rh*. *decoloratus*, *A*. *variegatum* and *H*. *elliptica*) were found on animals and environment in all the districts (Fig 2). The tick species variation per district was significant (χ^2^ = 32.88, *df* = 20, *p* = 0.035). Seventy-nine tick pools could not be fully identified because they contained larvae and nymphs with incomplete body parts.

### Prevalence of *Rickettsia* spp. in the tick pools

Calculated Maximum likelihood estimates (MLE) and Minimum infection rates (MIR) of *Rickettsia* spp. can be found in Table 1. The overall pool positivity rate was 24.6% (116/471) for all the districts, with the highest rate in Gulu of 30% and the lowest in Kasese with 14%. Livestock (cattle, goats, sheep, and pigs) had the highest pool positivity at 26.9% (95% CI 22.0, 31.9), followed by vegetation 23.1% (95% CI 6.9, 39.3) and companion animals, 19.7% (95% CI 13.1, 26.4). The one *Rh*. *decoloratus* tick pool obtained from a chicken was positive. The MLE for *Rickettsia* spp. by district is as follows: Gulu district had the highest MLE of 4.8% (95% CI 3.6, 6.2) with a corresponding MIR of 3.7% (95% CI 2.6, 4.8) while Kasese had the lowest MLE of 1.1% (95% CI 0.6, 2.0) with a MIR of 1.0% (95% CI 0.3, 1.7). In general, higher MLE values were obtained in districts in the northern region. Specifically, in Gulu district, all tick species (including nymphs) apart from *Rh*. *sanguineus* had high MLEs ranging from 2.8 to 23.8. *Rickettsia*-positive tick pools were from three genera, *Amblyomma* (48.8%), *Rhipicephalus* (24.2%), and *Haemaphysalis* (17.1%). *Amblyomma variegatum* had the highest MLE and MIR across tick species while other tick species had variable MLEs based on districts of collection (Table 1). This tick species also had the highest MLE values in all districts aside from Kasese and Kampala. In three districts, Gulu, Kampala, and Luwero, nymphs had MLE values ranging from 2.7 to 6.4.

**Table 1:** Maximum Likelihood Estimates (MLE) and Minimum Infection Rate (MIR) with corresponding 95% confidence intervals for detection rates of *Rickettsia* spp. in all tick pools.

District	Tick species	Positive pools (%)		Total ticks	MLE			MIR		
					Point	Low	High	Point	Low	High
**Gulu**	*A*. *variegatum*	9/14	(64%)	81	23.8	12.6	36.6	11.1	4.3	18.0
	*H*. *elliptica*	3/26	(12%)	93	3.5	1.2	9.3	3.2	0	6.8
	*Rh*. *appendiculatus*	10/43	(23%)	427	2.8	1.5	4.6	2.3	0.9	3.8
	*Rh*. *Decoloratus*	13/32	(41%)	312	6.1	3.5	9.4	4.2	2.0	6.4
	*Rh*. *Sanguineus*	0/1	(0%)	2	0	0	54.6	0	-	-
	Nymphs	9/30	(30%)	277	4.4	2.3	7.3	3.3	1.2	5.3
	**Total**	**44/146**	**(30%)**	**1192**	**4.8**	**3.6**	**6.2**	**3.7**	**2.6**	**4.8**
**Kasese**	*A*. *variegatum*	0/2	(0%)	2	0	0	65.8	0	-	-
	*H*. *elliptica*	0/9	(0%)	38	0	0	7.5	0	-	-
	*Rh*. *appendiculatus*	4/22	(18%)	271	1.6	0.6	3.3	1.5	0	2.9
	*Rh*. *Decoloratus*	4/18	(22%)	464	1.0	0.4	2.2	0.9	0	1.7
	Nymphs	0/6	(0%)	35	0	0	6.0	0	-	-
	**Total**	**8/57**	**(14%)**	**810**	**1.1**	**0.6**	**2.0**	**1.0**	**0.3**	**1.7**
**Kampala**	*A*. *variegatum*	2/2	(100%)	23	-	-	-	8.7	0	20.2
	*H*. *elliptica*	0/3	(0%)	7	0	0	28.0	0	-	-
	*Rh*. *appendiculatus*	1/20	(5%)	169	0.6	0.1	2.9	0.6	0	1.8
	*Rh*. *Decoloratus*	7/29	(24%)	461	1.7	0.8	3.1	1.5	0.4	2.6
	*Rh*. *Sanguineus*	0/2	(0%)	3	0	0	49.9	0	-	-
	Nymphs	3/11	(17%)	47	6.4	2.3	14.5	6.4	0	13.4
	**Total**	**13/67**	**(19%)**	**710**	**2.0**	**1.2**	**3.2**	**1.8**	**0.9**	**2.8**
**Jinja**	*A*. *variegatum*	4/8	(50%)	34	16.6	6.0	32.1	11.8	0.9	22.6
	*H*. *elliptica*	2/11	(18%)	138	2.3	0.5	7.7	1.5	0	3.4
	*Rh*. *appendiculatus*	1/15	(7%)	117	0.9	0.2	4.2	0.9	0	2.5
	*Rh*. *Decoloratus*	5/20	(25%)	183	3.0	1.3	6.0	2.7	0.4	5.1
	*Rh*. *Sanguineus*	0/1	(0%)	1	0	0	79.4	0	-	-
	Nymphs	0/7	(0%)	34	0	0	7.5	0	-	-
	**Total**	**12/62**	**(19%)**	**507**	**2.8**	**1.6**	**4.7**	**2.4**	**1.0**	**3.7**
**Luwero**	*A*. *variegatum*	5/15	(21%)	70	7.5	3.4	13.6	7.1	1.1	13.2
	*H*. *elliptica*	7/21	(44%)	135	6.9	3.2	12.8	5.2	1.5	8.9
	*Rh*. *appendiculatus*	15/44	(34%)	1232	1.6	1.0	2.3	1.2	0.6	1.8
	*Rh*. *Decoloratus*	8/34	(24%)	972	0.9	0.5	1.5	0.8	0.3	1.4
	Nymphs	4/25	(16%)	162	2.7	1.1	6.0	2.5	0	4.9
	**Total**	**39/139**	**(28%)**	**2571**	**1.8**	**1.4**	**2.3**	**1.5**	**1.0**	**2.0**
**All districts**	**Total**	**116/471**	**(25%)**	**5790**	**2.4**	**2.0**	**2.8**	**2.0**	**1.6**	**2.4**

### *Rickettsia* spp. identified by nucleotide sequences and phylogenetic analysis

**Fig 3:**
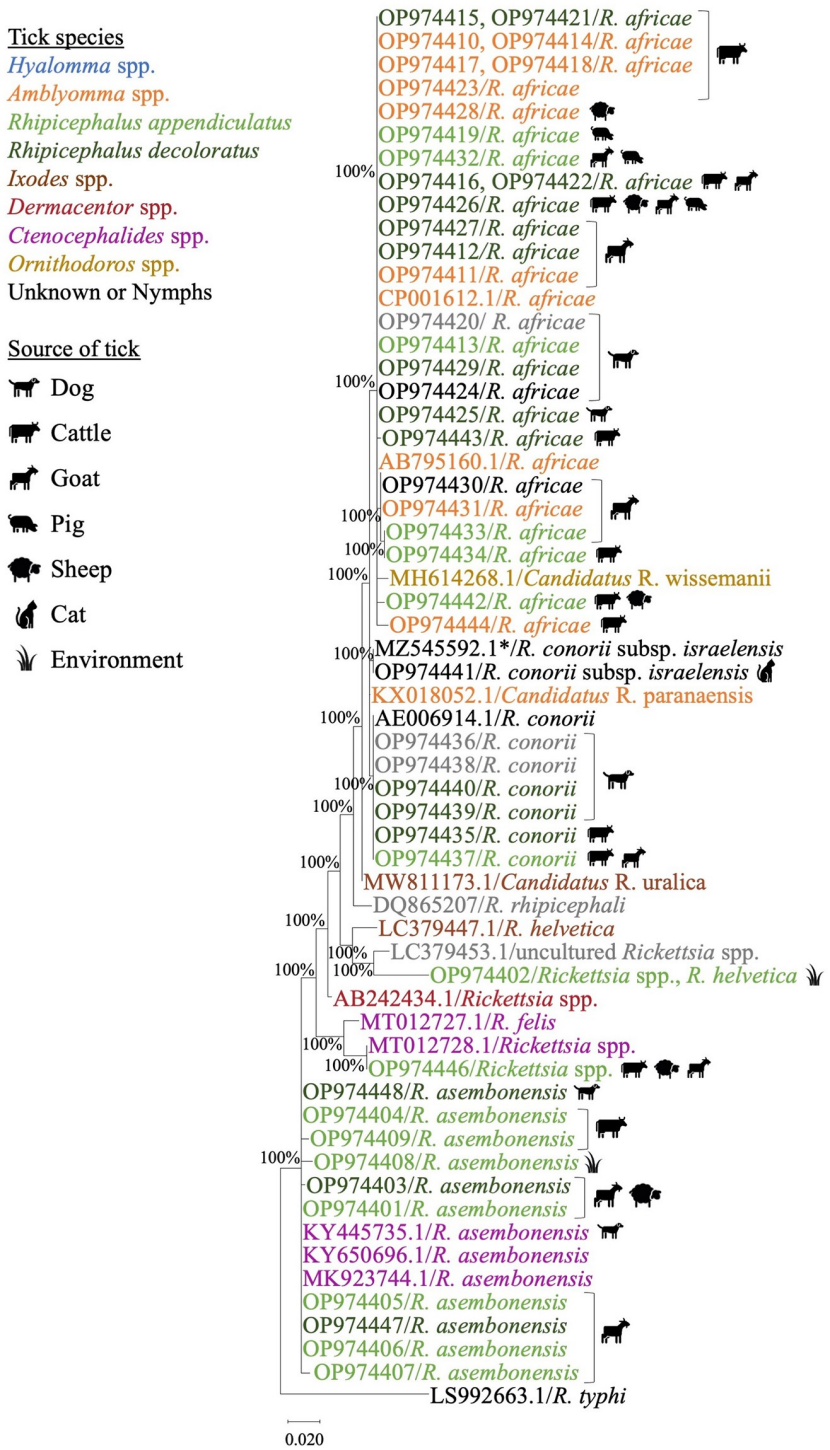
Maximum likelihood tree of the *17kDa Rickettsia* spp. gene using the Tamura-Nei model with sequences ranging from 192 to 426bp. Values less than 70% were excluded from the tree. The legend shows the tick species from which the Rickettsia spp. shown in this tree were detected. The legend shows the source of isolation by tick species and tick host by symbol. If multiple source icons appear next to an accession number, the pool of ticks came from more than one source. *One sample, MZ545592.1 was isolated from human serum and not from a tick. All GenBank accession numbers beginning with OP were sequenced in this study.

**Fig 4:**
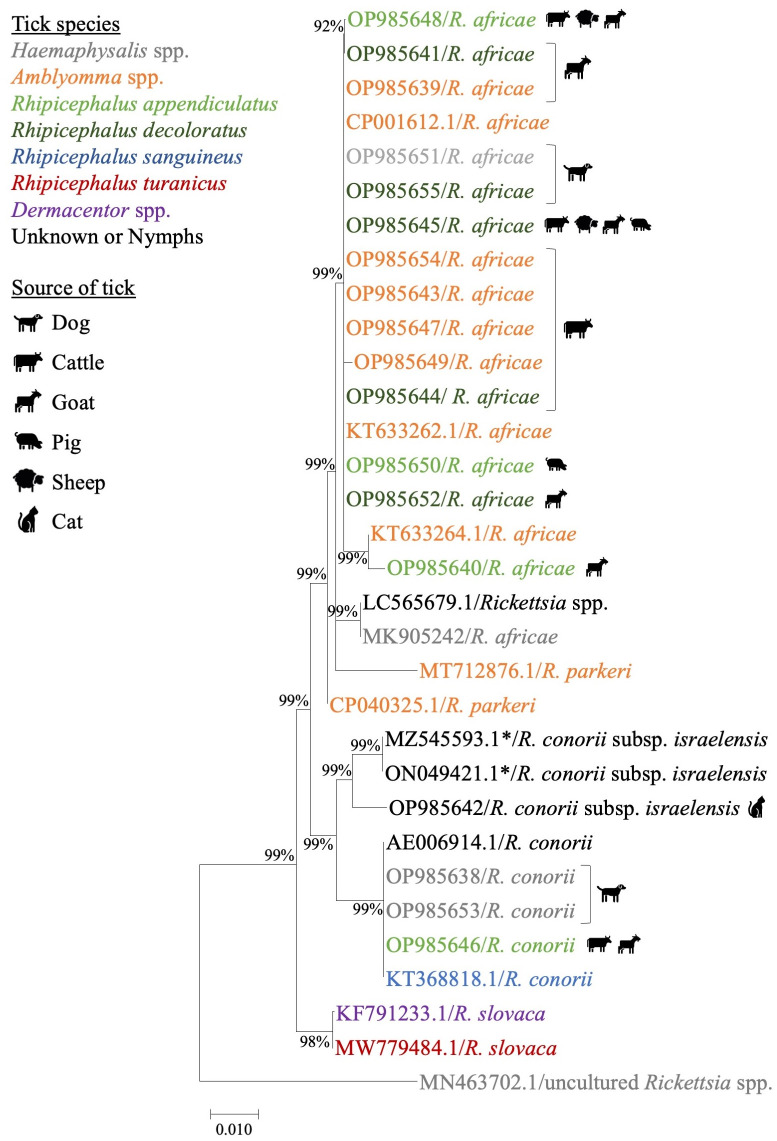
Maximum likelihood tree of the *ompA Rickettsia* spp. gene using the Tamura-Nei model with sequence lengths ranging from 545 to 590bp. Values less than 70% were excluded from the tree. The legend shows the tick species from which the Rickettsia spp. shown in this tree were detected. The legend shows the source of isolation by tick species and tick host by symbol. If multiple source icons appear next to an accession number, the pool of ticks came from more than one source. *One sample, MZ545592.1 was isolated from human serum and not from a tick. All GenBank accession numbers beginning with OP were sequenced in this study.

Of the 86 tick pools positive from *gltA* and *17kDa*, 48 pools of purified PCR amplicons were successfully sequenced. The nucleotide sequences obtained from *17kDa* (~539 base pairs) and *ompA* (~650 base pairs) were compared to those available on NCBI GenBank database by BLASTn analyses. Sequence identity and phylogenetic trees are presented in Table 2 and Figs 3 and 4. Using the *17kDa* and *ompA* genes, five *Rickettsia* spp. were identified from the tick pools: *R*. *africae*, *R*. *conorii*, *R*. *conorii* subsp. *israelensis*, *R*. *asembonensis*, and *R*. *helvetica*. The predominant *Rickettsia* spp. identified was *R*. *africae*, which was detected in four tick species (*A*. *variegatum*, *Rh*. *appendiculatus*, *Rh*. *decoloratus* and *H*. *elliptica)*, followed by *R*. *conorii* in three tick species (*Rh*. *appendiculatus*, *Rh*. *decoloratus*, and *H*. *elliptica*). *Rickettsia conorii* subsp. *israelensis* was identified in one nymph pool from a cat. *Rickettsia africae* were recovered from all animal types excluding cats, with *R*. *conorii* detected in ticks removed from cattle, goats, dogs, and a cat. *Rickettsia asembonensis* was found in livestock, grass, and a dog and *R*. *helvetica* was detected in *Rh*. *appendiculatus* collected from the environment.

**Table 2:** *Rickettsia* spp. identified with the corresponding GenBank accession numbers and identity to sequences on GenBank.

Gene	*Rickettsia* spp.	District	GenBank	Accession ID	Identity
*17kDa*	*R*. *africae*	Luwero	OP974408	MK923744.1	99.34%
		Luwero	OP974422	**MG515013**	100%
		Luwero	OP974434		99.55%
		Luwero	OP974443		99.10%
		Gulu	OP974411-OP974413, OP974418-OP974420, OP974423-OP974428, OP974432		100%
		Gulu	OP974442		99.55%
		Gulu	OP974445		98.77%
		Gulu	OP974433		99.55%
		Gulu	OP974429-OP974431		99.78%
		Kampala	OP974444		99.33%
		Kampala	OP974410		100%
		Jinja	OP974414-OP974416		100%
	*R*. *asembonensis*	Luwero	OP974421	**MG515013**	100%
		Luwero	OP974406*	MK923744.1	98.96%
		Luwero	OP974409		99.57%
		Kasese	OP974401		99.78%
		Kasese	OP974404		100%
		Kampala	OP974447		96.47%
		Gulu	OP974407	KY445736.1	99.40%
		Kasese	OP974403		98.80%
		Kampala	OP974405		99.00%
		Kampala	OP974448		100%
	*R*. *conorii*	Luwero	OP974436-OP974438, OP974440, OP974435	AE006914.1	100%
	*R*. conorii subsp. *Israelensis*	Gulu	OP974441	**MZ545592.1**	100%
	*Rickettsia* spp.	Luwero	OP974446	MT012728.1	100%
	uncultured *Rickettsia* spp./*Rickettsia helvetica*	Kasese	OP974402	LC379453.1	96.07%
*ompA*	*R*. *africae*	Luwero	OP985645, OP985647-OP985648	CP001612.1	100%
		Gulu	OP985649		99.51%
		Gulu	OP985639, OP985641, OP985650-OP985652, OP985654-OP985655		100%
		Jinja	OP985643-OP985644		100%
	*R*. *conorii* subsp. *Israelensis*	Gulu	OP985642	**MZ545593.1**	98.52%
	*R*. *conorii*	Luwero	OP985638, OP985646, OP985653	AE006914.1	100%

The sequences in bold are human cases from Brazil (MG515013) and Iran (MZ545592.1, MZ545593.1). The length of the *17kDa* sequences were 326-514bp and *ompA* sequences were 545-609bp.

*Sequence was 202bp

Ticks collected from all livestock species had similar sequences for *R*. *africae*, *R*. *conorii*, and *R*. *asembonensis* based on the *17kDa* gene. The two sequences from the environmentally collected ticks were unique compared to previously published sequences. Based on the *17kDa* sequences from this study, tick pools from Gulu, Jinja, Luwero and Kampala had identical homology with *R*. *africae* strain PELE, which was isolated from a human traveller in Brazil. *Rickettsia conorii* subsp. *israelensis* detected in the Gulu district matched fatal human case detected in Iran. *Rickettsia asembonensis 17kDa* sequences obtained from ticks in this study were highly similar to that from flea samples from South America. The *R*. *helvetica* sequence from this study was unique compared to sequences published on GenBank. The *ompA* sequence comparison revealed *R*. *africae* from this study were identical to a sequence isolated from *A*. *variegatum* ticks from Ethiopia and Benin. Additionally, the *R*. *conorii* sequences were identical to one from a *Rh*. *sanguineus* tick from a dog in Romania.

## Discussion

A relatively high pool positivity rate (24.6%) for *Rickettsia* spp. was detected in this study. This *Rickettsia* spp. positivity is comparable to a similar study in Kenya that demonstrated 25% *Rickettsia* spp. prevalence in tick pools collected from livestock and camels in dispersed pastoral communities [36]. The highest positive pool detection rates among tick species in our study were in *A*. *variegatum* (48.8%), *Rh*. *decoloratus* (27.8%) and *Rh*. *appendiculatus* (21.6%). These ticks feed predominantly on cattle, sheep, goats and large wild ruminants [20]. There is evidence of up to 97% prevalence of *R*. *africae* in *A*. *variegatum* collected from cattle in Eastern parts of Uganda [32]. Similar to other findings from sub-Saharan Africa, our study confirms that *Rickettsia* spp. are likely present across Uganda where animals reside, posing a risk to over half of the Ugandan population that derive their livelihoods from animals [27,37–39]. *Rickettsia*-positive ticks were found in every district, with higher MLEs and pool-positivity rates observed in northern and eastern regions of Uganda suggesting potential hotspots for *Rickettsia* spp. infections that need to be further investigated.

The relative variation in infection rates by district could be explained by differences in livestock populations and intensity of acaricide use. Farms in central and western Uganda are more likely to have dairy cattle and use more acaricides compared to livestock farms in northern and eastern Uganda, which predominantly have indigenous cattle [40–41]. Acaracide usage could account for the greater tick-species diversity in Gulu as opposed to the other districts. Areas that spray acaracides on their livestock can reduce the number and diversity of susceptible tick species because of selective pressure. However, tick species that are resistant to acaricides, *Amblyomma* spp. and *Rhipicephalus* spp., are likely to persist in Uganda [42]. *Amblyomma* spp. are the major vector for *R*. *africae* in sub-Saharan Africa and are likely harboring many *Rickettsia* pathogens in northern Uganda with the highest MLE result. Potential resistance in this species may contribute to the maintenance of the highly prevalent *R*. *africae* in the northern region. *Rhipicephalus* ticks are the most prevalent in Uganda on livestock and were the most common tick genus collected from every district [41]. There have been detections of acaracide resistant *Rhipicephalus* spp. in northern Uganda brought by livestock movements [43]. The continued development of acaracide resistance in this genus would pose a large threat in Uganda as all *Rickettsia* spp. detected in this study were present Livestock trading could lead to the movement of ticks, potentially with acaracide resistance, across district or country borders.

While livestock rearing increases the risk for SFG rickettsia, another big industry in Uganda at risk for tick-borne diseases is tourism. One major tourism area in the Kasese district, Queen Elizabeth National park, has an estimated 34,000 visitors annually [44]. This environment is conducive to wildlife and domestic encounters increasing the risk for human contact with infected ticks. Specifically, ATBF has been reported in a Slovenian traveller returning from southwestern Uganda [14]. Surprisingly, Kasese district has the lowest MLE, given the large number of tourists visiting the area. Additional sampling should be done in this district to understand the risk of *Rickettsia* spp. to tourists as *R*. *africae* was not detected in this region, likely because only two *A*. *variegatum* ticks were collected. Travelers should be aware of potential illnesses associated with pathogens present in the region.

Multiple *Rickettsia* sequences (*R*. *africae* and *R*. *conorii*) from this study had high homology to sequences causing human illness in Brazil and Iran emphasizing the importance of monitoring these pathogens in Uganda. The *R*. *africae* strain, PELE, caused ATBF leading to hospitalization in a traveler from South Africa and was identical to *17kDa* sequences from this study from multiple districts [11]. Of concern is the *R*. *conorii* subsp. *israelensis* sequence from Gulu district that matched a fatal human case from Iran [45]. The discovery of these pathogens in the most abundant tick species on livestock [41] poses a high risk of potential illness to Ugandans.

As the first report of *R*. *africae* in Uganda in *Rh*. *appendiculatus* and *Rh*. *decoloratus* in all collection districts aside from Kasese, there is a need for more surveillance of *Rickettsia* spp. among *Rhipicephalus* ticks. Especially because this study confirmed these two *Rhipicephalus* spp. are the most abundant livestock ticks in Uganda and they are the most multi-acaricide resistant ticks on animal farms [20,30]. This study also presents the first detection of *R*. *conorii* in *Rh*. *appendiculatus* and *Rh*. *decoloratus* ticks in Uganda, which could lead to MSF, especially since a common transmission route is contact with domestic animals [22]. Another causative agent of febrile illness, *R*. *helvetica*, was found for the first time in Uganda in *Rh*. *appendiculatus* and was unique to other published sequences. Interestingly, this tick was collected from vegetation and not from livestock. *Rickettsia asembonensis* was detected in two tick species, *Rh*. *appendiculatus* and *Rh*. *decoloratus*, but it is mostly flea borne. It has occasionally been detected in ticks with limited information about its pathogenicity in humans [46]. Additional studies on *Rhipicephalus* genera would be beneficial to understanding the scope of these *Rickettsia* spp. in these ubiquitous vectors across Uganda.

### Limitations

Ticks were collected from five districts in Uganda to represent the four regions. However, the results from these districts may not be representative of *Rickettsia* spp. found within the entire respective region, so careful consideration was taken when extrapolating the results. Additionally, a limited number of ticks (64/5790) were collected from the vegetation and analyzed in 26/471 pools so minimal environmental conclusions were made in this study. Ticks were identified solely using morphology thus limiting the confidence of species identification. By pooling ticks, the MLE was immeasurable when 100% of tick pools were positive and MIR was immeasurable when 0% of tick pools were positive and this was noted in Table 2 using dashes (-). The PCR targets were designed for one species, *Rickettsia*, so co-infection was not assessed.

## Conclusions

This is the first major study using targeted gene sequencing for *Rickettsia* spp. covering diverse ecological zones of Uganda. The detection of *Rickettsia* spp. in every surveyed district and in multiple tick species highlights the need to monitor the threat of rickettsial disease in these regions and develop rapid diagnostic tests. This was the first detection of the ISF agent in ticks in Uganda and the first identification of ATBF and MSF causative agents in *Rh*. *appendiculatus and Rh*. *decoloratus* ticks in Uganda. Clinicians must be informed of circulating *Rickettsia* spp. endemic to Uganda to timely and effectively detect, treat, and prevent human illness. Further tick-borne pathogen surveillance and seroprevalence studies are essential in Uganda to further characterize *Rickettsia* spp. which threaten Ugandans, travelers, and public health.

## References

[1] Raoult D, Roux V. Rickettsioses as paradigms of new or emerging infectious diseases. Clin Microbiol Rev. 1997;10(4):694–719. doi: 10.1128/CMR.10.4.694 9336669 PMC172941

[2] Parola P, Paddock CD, Raoult D. Tick-borne rickettsioses around the world: emerging diseases challenging old concepts. Clin Microbiol Rev. 2005 Oct 1;18(4):719–56. Available from: http://www.ncbi.nlm.nih.gov/pubmed/16223955 doi: 10.1128/CMR.18.4.719-756.2005 16223955 PMC1265907

[3] Walker HD. Rickettsiae and Rickettsial Infections: The Current State of Knowledge. Clinical Infectious Diseases. 2007 Jul 15;45(Supplement_1):S39–44. doi: 10.1086/518145 17582568

[4] Prabhu M, Nicholson WL, Roche AJ, Kersh GJ, Fitzpatrick KA, Oliver LD, et al. Q Fever, Spotted Fever Group, and Typhus Group Rickettsioses Among Hospitalized Febrile Patients in Northern Tanzania. Oxford University Press. 2011;53(4):e8–15. doi: 10.1093/cid/cir411 21810740 PMC3148261

[5] Thiga JW, Mutai BK, Eyako WK, Ng’Ang’A Z, Jiang J, Richards AL, et al. High seroprevalence of antibodies against spotted fever and scrub typhus bacteria in patients with febrile illness, Kenya. Emerg Infect Dis. 2015;21(4). doi: 10.3201/eid2104.141387 25811219 PMC4378494

[6] Parola P, Paddock CD, Socolovschi C, Labruna MB, Mediannikov O, Kernif T, et al. Update on Tick-Borne Rickettsioses around the World: A Geographic Approach. Clin Microbiol Rev. 2013 Oct;26(4):657–702. doi: 10.1128/CMR.00032-13 24092850 PMC3811236

[7] Mayxay M, Castonguay-Vanier J, Chansamouth V, Dubot-Pérès A, Paris DH, Phetsouvanh R, et al. Causes of non-malarial fever in Laos: A prospective study. The Lancet Global Health. 2013;1(1):46–54. doi: 10.1016/S2214-109X(13)70008-1 24748368 PMC3986032

[8] Abhilash KPP, Jeevan JA, Mitra S, Paul N, Murugan TP, Rangaraj A, et al. Acute Undifferentiated Febrile Illness in Patients Presenting to a Tertiary Care Hospital in South India: Clinical Spectrum and Outcome. Journal of global infectious diseases. 2016;8(4):147–54. doi: 10.4103/0974-777X.192966 27942194 PMC5126753

[9] Weinberger M, Keysary A, Sandbank J, Zaidenstein R, Itzhaki A, Strenger C, et al. Fatal Rickettsia conorii subsp. israelensis infection, Israel. Emerging Infectious Diseases. 2008;14(5):821–4. doi: 10.3201/eid1405.071278 18439372 PMC2600240

[10] Sekeyová Z, Danchenko M, Filipčík P, Fournier PE. Rickettsial infections of the central nervous system. PLoS Neglected Tropical Diseases. 2019;13(8):1–18. doi: 10.1371/journal.pntd.0007469 31465452 PMC6715168

[11] Angerami RN, Krawczak FS, Nieri-Bastos FA, Santos F, Medorima C, Resende MR, et al. First report of African tick-bite fever in a South American traveler. SAGE Open Medical Case Reports. 2018;6:2050313X1877530. 10.1177/2050313X18775301 PMC5956635 29796268

[12] Ericsson CD, Jensenius M, Fournier PE, Raoult D. Rickettsioses and the International Traveler. Clinical Infectious Diseases. 2004 Nov 15;39(10):1493–9. doi: 10.1086/425365 15546086

[13] Eldin C, Parola P. Update on tick-borne bacterial diseases in Travelers. Current Infectious Disease Reports. 2018;20(17). doi: 10.1007/s11908-018-0624-y 29789953

[14] Bogovic P, Lotric-furlan S, Korva M, Avsic-zupanc T. African Tick-Bite Fever in Traveler Returning to Slovenia from Uganda. Emerg Infect Dis. 2016;22(10):1848–9. doi: 10.3201/eid2210.160650 27648844 PMC5038416

[15] Stewart AG, Stewart AGA. An update on the laboratory diagnosis of rickettsia spp. Infection. Pathogens. 2021;10(10):1–11. doi: 10.3390/pathogens10101319 34684267 PMC8541673

[16] Kovácová E, Kazár J. Rickettsial diseases and their serological diagnosis. Clinical laboratory. 2000;46(5–6):239–45. 10853230

[17] Paris DH, Dumler JS. State of the art of diagnosis of rickettsial diseases: The use of blood specimens for diagnosis of scrub typhus, spotted fever group rickettsiosis, and murine typhus. Curr Opin Infect Dis. 2016;29(5):433–9. doi: 10.1097/QCO.0000000000000298 27429138 PMC5029442

[18] Ghai RR, Thurber MI, El Bakry A, Chapman CA, Goldberg TL. Multi-method assessment of patients with febrile illness reveals over-diagnosis of malaria in rural Uganda. Malaria Journal. 2016 Dec 7;15(1):460. doi: 10.1186/s12936-016-1502-4 27604542 PMC5015337

[19] Kabi F, Masembe C, Muwanika V, Kirunda H, Negrini R. Geographic distribution of non-clinical Theileria parva infection among indigenous cattle populations in contrasting agro-ecological zones of Uganda: Implications for control strategies. Parasites and Vectors. 2014;7(1):1–9. doi: 10.1186/1756-3305-7-414 25175844 PMC4261563

[20] Byaruhanga C, Collins NE, Knobel D, Kabasa W, Oosthuizen MC. Endemic status of tick-borne infections and tick species diversity among transhumant zebu cattle in Karamoja Region, Uganda: Support for control approaches. Veterinary Parasitology: Regional Studies and Reports. 2015;1–2:21–30. doi: 10.1016/j.vprsr.2015.11.001 31018404

[21] Corrigan J, Marion B, English J, Eneku W, Weng JL, Rugg M, et al. Minimal Rickettsial Infection Rates and Distribution of Ticks in Uganda: An Assessment of the Seasonal Effects and Relevance to Tick-Borne Disease Risk in East Africa. Journal of Medical Entomology. 2022;(Xx):1–8. 36321534 10.1093/jme/tjac166

[22] Onyiche TE, Labruna MB, Saito TB. Unraveling the epidemiological relationship between ticks and rickettsial infection in Africa. Frontiers in Tropical Diseases. 2022;3(September):1–29.

[23] Spernovasilis N, Markaki I, Papadakis M, Mazonakis N, Ierodiakonou D. Mediterranean spotted fever: Current knowledge and recent advances. Tropical Medicine and Infectious Disease. 2021;6(4). doi: 10.3390/tropicalmed6040172 34698275 PMC8544691

[24] Proboste T, Kalema-Zikusoka G, Altet L, Solano-Gallego L, Fernández De Mera IG, Chirife AD, et al. Infection and exposure to vector-borne pathogens in rural dogs and their ticks, Uganda. Parasite and Vectors. 2015;8(1):1–9. doi: 10.1186/s13071-015-0919-x 26043771 PMC4460633

[25] Olwoch JM, Reyers B, Engelbrecht FA, Erasmus BFN. Climate change and the tick-borne disease, Theileriosis (East Coast fever) in sub-Saharan Africa. Journal of Arid Environments. 2008;72(2):108–20.

[26] Khatchikian CE, Prusinski M, Stone M, Bryon Backenson P, Wang IN, Levy MZ, et al. Geographical and environmental factors driving the increase in the Lyme disease vector Ixodes scapularis. Ecosphere. 2012;3(10):1–18. doi: 10.1890/ES12-00134.1 24371541 PMC3872055

[27] Waiswa D, Günlü A, Mat B. Development opportunities for livestock and dairy cattle production in Uganda: A Review. Res J Agriculture and Forestry Sci International Science Community Association. 2021;9(1):18–24.

[28] Boehnke D, Brugger K, Pfäffle M, Sebastian P, Norra S, Petney T, et al. Estimating Ixodes ricinus densities on the landscape scale. International Journal of Health Geographics. 2015;14(1):1–12. doi: 10.1186/s12942-015-0015-7 26272596 PMC4536605

[29] Public Health Ontario. Active Tick Dragging: Standard Operating Procedure. Queen’s Printer for Ontario. 2015;(11). https://www.publichealthontario.ca/en/eRepository/Active_tick_dragging_SOP.pdf

[30] Walker AR, Bouattour A, Camicas JL, Estrada-peña A, Horak IG, Latif A a, et al. Ticks of domestic animals in Africa: a guide to identification of species. The University of Edinburgh. 2003. p.227.

[31] Stenos J, Graves SR, Unsworth NB. A highly sensitive and specific real-time PCR assay for the detection of spotted fever and typhus group Rickettsiae. Am J Trop Med Hyg [Internet]. 2005;73(6):1083–5. 16354816

[32] Nakao R, Qiu Y, Igarashi M, Magona JW, Zhou L, Ito K, et al. High prevalence of spotted fever group rickettsiae in Amblyomma variegatum from Uganda and their identification using sizes of intergenic spacers. Ticks Tick Borne Dis. 2013;4(6):506–12. doi: 10.1016/j.ttbdis.2013.07.001 24331642

[33] Noh Y, Lee YS, Kim HC, Chong ST, Klein TA, Jiang J, et al. Molecular detection of Rickettsia species in ticks collected from the southwestern provinces of the Republic of Korea. Parasit Vectors [Internet]. 2017;10(1):1–10. Available from: 10.1186/s13071-016-1955-x 28069059 PMC5223493

[34] Kumar S, Stecher G, Li M, Knyaz C, Tamura K. MEGA X: Molecular evolutionary genetics analysis across computing platforms. Mol Biol Evol. 2018;35(6):1547–9. doi: 10.1093/molbev/msy096 29722887 PMC5967553

[35] QGIS.org. QGIS Geographic Information System. QGIS Association. [Internet]. 2022 [cited 2023 Feb 25]. https://www.qgis.org/en/site/

[36] Koka H, Sang R, Kutima HL, Musila L, Macaluso K. The detection of spotted fever group rickettsia DNA in tick samples from pastoral communities in Kenya. Journal of Medical Entomology. 2017;54(3):774–80. doi: 10.1093/jme/tjw238 28073909 PMC5850802

[37] Adjou Moumouni PF, Terkawi MA, Jirapattharasate C, Cao S, Liu M, Nakao R, et al. Molecular detection of spotted fever group rickettsiae in Amblyomma variegatum ticks from Benin. Ticks and Tick-borne Diseases. 2016;7(5):828–33. doi: 10.1016/j.ttbdis.2016.03.016 27150592

[38] Tomassone L, De Meneghi D, Adakal H, Rodighiero P, Pressi G, Grego E. Detection of Rickettsia aeschlimannii and Rickettsia africae in ixodid ticks from Burkina Faso and Somali Region of Ethiopia by new real-time PCR assays. Ticks and Tick-borne Diseases. 2016;7(6):1082–8. doi: 10.1016/j.ttbdis.2016.09.005 27641952

[39] Nakao R, Qiu Y, Salim B, Hassan SM, Sugimoto C. Molecular Detection of Rickettsia africae in Amblyomma variegatum Collected from Sudan. Vector-Borne and Zoonotic Diseases. 2015;15(5). doi: 10.1089/vbz.2014.1748 25988442 PMC4449622

[40] Vudriko P, Okwee-Acai J, Byaruhanga J, Tayebwa DS, Okech SG, Tweyongyere R, et al. Chemical tick control practices in southwestern and northwestern Uganda. Ticks and Tick-borne Diseases. 2018;9(4):945–55. doi: 10.1016/j.ttbdis.2018.03.009 29606621

[41] Kasaija PD, Estrada-Peña A, Contreras M, Kirunda H, de la Fuente J. Cattle ticks and tick-borne diseases: a review of Uganda’s situation. Ticks and Tick-borne Diseases. 2021;12(5). doi: 10.1016/j.ttbdis.2021.101756 34134062

[42] Vudriko P, Okwee-Acai J, Tayebwa DS, Byaruhanga J, Kakooza S, Wampande E, et al. Emergence of multi-acaricide resistant Rhipicephalus ticks and its implication on chemical tick control in Uganda. Parasites and Vectors. 2016;9(1). doi: 10.1186/s13071-015-1278-3 26727991 PMC4700616

[43] Selby R, Bardosh K, Picozzi K, Waiswa C, Welburn SC. Cattle movements and trypanosomes: Restocking efforts and the spread of Trypanosoma brucei rhodesiense sleeping sickness in post-conflict Uganda. Parasites and Vectors. 2013;6(1):1–12. doi: 10.1186/1756-3305-6-281 24289452 PMC3851531

[44] Dunwiddie L, Shaw RT. Balancing Conservation and Development: A Case Study of Economic Efficiency in Queen Elizabeth National Park, Uganda (2013). Independent Study Project (ISP) Collection. 1691. https://digitalcollections.sit.edu/isp_collection/1691

[45] Esmaeili S, Latifian M, Khalili M, Farrokhnia M, Stenos J, Shafiei M, et al. Fatal Case of Mediterranean Spotted Fever Associated with Septic Shock, Iran. Emerging Infectious Diseases. 2022;28(2):485–8. doi: 10.3201/eid2802.211023 35076374 PMC8798672

[46] Kocher C, Morrison AC, Leguia M, Loyola S, Castillo RM, Galvez HA, et al. Rickettsial Disease in the Peruvian Amazon Basin. PLoS Negl Trop Dis. 2016;10(7):1–13. doi: 10.1371/journal.pntd.0004843 27416029 PMC4944934

